# The Vindication of Water

**Published:** 1895-12-07

**Authors:** 


### The Vindication of Water.

The ill-repute into which cold water had fallen in the 16th century applied, according to medical phraseology, either extra or intus, was attributed with some show of reason by Dr. Floyer, its first champion, to the Reformation. So long as the old sacred shrines, often situated near springs, held their supernatural character, the custom of immersion in the holy water held good, and "the common people attributed all their cures to the merit of that saint and their own devotion, which was due to the physical virtue of cold springs and God's blessing on the natural use of them." The civil wars, too, and the consequent unquiet state of the country, were responsible for the total disuse of many famous waters. But gradually, and in the teeth of the strongest opposition from many of the learned, the reputation of certain English springs began to revive, and the custom of water drinking which, according to Tobias Whitaker, had hitherto been permitted "only upon such strict and warie terms and circumstances as rarely or never will concurre," began to gain ground.

The first revival of the fashion of frequenting mineral waters grew out of the great popularity of the German Spa. The doctors began to grow incensed at the departure of all their wealthy patients to a foreign cure, and the opportunity which a stay at the Spa afforded "for all wished means of private converse with priests and Jesuits" was quoted as a grave source of national danger. One after another the old mineral waters of Bath, Tunbridge, and Buxton were set in order, and their virtues set forth in cheap tractates relating a ceaseless series of wonderful cures. New springs were discovered, notably the Tuit Well at Knaresborough, warranted to have "a perfect Spa relish," and the Warwickshire waters of Newnam Regis. But the taking of mineral, or indeed any kind of water, was still considered a rash and eccentric proceeding, calculated "to corrupt them that are sound, to weaken those that are strong, to hasten age in those that are young, and, in conclusion, to strangle and swallow up all in death; such have been the effects of mineral wells and fountains." The use of the waters, even by those who dared to advocate them, was safeguarded by many superstitious observances relating to times and seasons. William Bayley, a strong partisan of the waters, gravely argues the question whether the same may be ministered in leap year, and reaches the conclusion, "so for my part I would not rashly counsel any to use them in the leap years unless great cause do urge the use of them."

Dr. Floyer (1649-1734), who from the beginning of his career set himself to restore cold water to its natural place as a therapeutic agency, was remarkable among the physicians of the day no less for his scientific attainments and originality of thought than for his deep sense of religion and of the intimate connection between the diseases of the soul and body.

To judge from the controversies which grew up round the subject, Dr. Floyer never succeeded in making the use of cold water really popular. He was too far in advance of his age. His denunciations of the pet vices of the day won him bitter enemies, and the rule of life which he enforced on his patients was far too simple and natural to win many adherents in a period of artificiality and excitement. He inveighed in round terms against the luxurious habits which prevailed "without any such intermission as the Church advises in Lent and fasting days," and was specially hard on "the vicious diet or regimen of women, who are taught to drink not only hot wines and strong liquors, chocolate, coffee, tea, from their youth; they are oft used to strong broths, high sauces and pickles, oysters, anchovies, herrings, mushrooms, strong pottages, and meat full of raw blood." He attributes "all rheumatisms, defluctions, intermittent fevers, &c., to the late practice of drinking hot liquors, and the pernicious use of flannel and woollen shirts next to the skin, which always keeps the pores of the skin too open for the climate we live in." However much experience may have modified the judgment of modern practitioners with respect to such enormities as the wearing of flannel and the drinking of tea and coffee, few will be now found to question the wisdom of the doctor's injunctions to avoid down beds and sitting by the fire, and to bathe in cold water, live much in the fresh air, and drink mainly water or mild beer in preference to the strongly spiced and heating wines of the period. But to those accustomed to a soft style of living, such orders met with scant favour. The arguments by which Floyer enforced the benefits of cold water need not here be recapitulated. Such as were not derived from the ancients were drawn from his own experience, supported by some sensible remarks on the part played by skin action in the economy of the human body.

He was specially successful in dealing with children. He protested against the "hot regimen," which produced "a soft flaccid flesh and porous skin," and which was, in fact, responsible for the death of all Queen Anne's children, and he became at length so strong an advocate for the cold bath that he wrote a treatise to prove conclusively that the rise of rickets in England, which he places about the year 1620, coincided with the final abolition of the Trine immersion in baptism, and to urge the restoration of this time-honoured custom as the best method of combating the disease. He urged that rickets were unknown in Wales, where baptism by immersion was still common, and where the nurses "ordinarily wash their children in cold water every day from their birth till they are three-quarters of a year old."

In later life Dr. Floyer, though sometimes guarding himself against the accusation of making water into a panacea, confessed there were few, if any, disorders for which it did not constitute the best remedy. He built a cold bath at Lichfield, and actually procured the revival in his neighbourhood of baptism by immersion. If he did not inaugurate hydropathy under its modern scientific aspects, he redeemed his favourite element from centuries of neglect and contempt, and restored the water drinker to public esteem. The water drinkers, he declared, "are temperate in their actions, prudent and ingenious, and they live safe from those diseases which affect the head, such are apoplexies, palsies, pain, blindness, deafness, gout, convulsions, trembling, madness."

